# Utility of mobility testing in early ADRD detection

**DOI:** 10.1002/alz.093026

**Published:** 2025-01-03

**Authors:** Magdalena I Tolea, Amie Rosenfeld, Lilah M Besser, James E Galvin

**Affiliations:** ^1^ University of Miami Miller School of Medicine, Boca Raton, FL USA; ^2^ University of Miami, Boca Raton, FL USA

## Abstract

**Background:**

Gait and balance deficiencies may be important indicators of cognitive impairment, distinguishing dementia from normal cognition (NC). It is unclear whether this extends to pre‐dementia stages of disease. Study goals were to: assess patterns of mobility across early stages of disease and identify specific measures that distinguish individuals with subjective cognitive impairment (SCI) and mild cognitive impairment (MCI).

**Method:**

We assessed whether mobility differentiates SCI (n = 35) from NC (n = 78) and MCI (n = 41). Using ANCOVA, we compared diagnostic groups on global (mini‐PPT; TUG) and individual (gait speed (GS), flexibility, balance, step length, single‐leg support) measures of mobility, controlling for age and baseline physical activity levels. Group separation was evaluated with logistic regression. Group differences in mobility while distinguishing between MCI etiology (AD vs non‐AD) and relationships between mobility and MRI cortical atrophy score (CAS) were analyzed and results validated against biomarker‐confirmed diagnoses (true controls/preclinical/MCI).

**Result:**

Participants were 69±9yr old, highly educated (16±3y), mostly non‐Hispanic White (77%), and performed within normal parameters for mobility. Mobility performance decreased with disease stage (MCI>SCI>NC). All mobility measures distinguished MCI from NC (p<0.01). Dual task mobility distinguished MCI from SCI (e.g., OR_TUG_ = 1.24, 95%CI:1.055‐1.457). AD vs non‐AD etiology accounted for most group differences in mobility. Higher mini‐PPT score was linked to lower odds of SCI vs NC (OR = 0.705 (0.543‐0.916)) stemming from significantly poorer balance in SCI (β = ‐0.3±0.1, p = 0.041). Lower GS explained MCI differences from NC (β = ‐0.1±0.03, p = 0.003) and SCI (β = ‐0.1±0.04, p = 0.027). Results were not explained by differences in age and physical activity. Small‐to‐moderate correlations between CAS and mobility (e.g., r_TUG dual_ = 0.34, p<0.001) were found. In analyses using biomarker‐confirmed diagnoses, mobility was lower in MCI vs true controls (all p<0.01) and vs preclinical disease (p<0.05), with a trend for preclinical vs true controls (p = 0.054) validating findings on clinical diagnoses.

**Conclusion:**

Mobility parameters can distinguish NC from SCI and MCI highlighting: 1) dual task testing and subtle changes in balance help detect early cognitive impairments; 2) changes in GS mark more significant cognitive impairment. Mobility testing may be an important tool in early detection of ADRD in the clinical setting and can help identify targets for early intervention.

